# The Ocular Biometry of Adult Cataract Patients on Lifeline Express Hospital Eye-Train in Rural China

**DOI:** 10.1155/2015/171564

**Published:** 2015-10-05

**Authors:** Xiaoguang Cao, Xianru Hou, Yongzhen Bao

**Affiliations:** Peking University People's Hospital, Ophthalmology Department, Key Laboratory of Vision Loss and Restoration, Ministry of Education, Beijing Key Laboratory for the Diagnosis and Treatment of Retinal and Choroid Diseases, Beijing 100044, China

## Abstract

*Aims*. To describe and explore the distribution of ocular biometric parameters of adult cataract patients in rural China.* Methods*. Three Lifeline Express Hospital Eye-Train missions of Peking University People's Hospital in China were chosen. 3828 adult cataract patients aged 29 to 88 years with axial length (AL) less than 27.0 mm were enrolled. The ocular biometry including visual acuity (VA), intraocular pressure, AL, corneal power (*K*1 and *K*2), and corneal endothelial counting (CEC) were collected and analysis. Corneal radius (CR) was calculated from the corneal power.* Results*. The participants in Zhoukou of these three missions had the worse preoperative VA (*p* < 0.001), the lowest *K*1 (*p* < 0.001), *K*2 (*p* < 0.001), and *K* (*p* < 0.001) and the highest |*K*1 − *K*2| (*p* < 0.001), moreover AL/CR more closely to 3.0. The AL, |*K*1 − *K*2|, and AL/CR were normally distributed. But the *K*1, *K*2, *K*, and CEC were not normal distributions. Except *K*1, all parameters were positively skewed and peaked.* Conclusion*. Our study provides normative ocular biometry in a large, representative rural Chinese population. The AL is normally distributed with a positive skew and big kurtosis. The corneal powers are not normal distribution. The corneal astigmatism might have a significant effect on the visual acuity.

## 1. Introduction

According to China's Ministry of Health, China has approximately 4 million cataract victims, with 500,000 new cases being diagnosed each year [1]. As a developing country, especially in rural China, poverty and limited access to health care, due to the uneven distribution of health care sources, can make it very difficult for these people to obtain proper treatment [2]. Cataract surgical rate (CSR) is still very low in rural China. Moreover, a lot of these cataract surgeries were charge-free. Lifeline Express Hospital Eye-Train (LEHET), the first charge-free cataract surgery project founded in 1997, is a quite important way to restore vision for the low-income rural people in China.

Independent of cost or other factors, the first expectation from surgeons and patients is good postoperative visual outcomes. To meet these expectations, attention to accurate biometry measurements is critical [3]. The biometry is indispensable to the surgeons and patients as it might indicate the prognosis and safety of the coming operation. In the biometric parameters, axial length (AL) and corneal curvature are the most important. However, the distribution and determinants of AL have been assessed in only a few population-based studies of older persons [4–10], of which there is still no study of rural Chinese population, especially in Middle China, having cities with extremely long history.

In 2011 and 2012, our hospital (Peking University People's Hospital, PUPH) had three missions of LEHET in Middle China. In this study, we explored the biometric parameters of adult cataract patients who had cataract surgeries on LEHET in these missions and all were rural people.

## 2. Methods

### 2.1. Recruitment of Patients, Preoperative Assessment, and Exclusion Criteria

Our hospital, PUPH, had four missions of LEHET, Zhoukou in Henan province and Songyuan in Jilin province in 2011, Yuncheng in Shanxi province and Sanmenxia in Henan province in 2012. The sites were selected by the office of LEHET, and they were blind to our hospital before the mission start. Songyuan in Jilin province was excluded from this study as the incompleted data. Yuncheng (N 35.03; E 111.01; altitude: 369.53 m) in Shanxi province and Zhoukou (N 33.62; E 114.66; altitude: 50.50 m) in Henan province have thousands of years of history. Most residents in these two cities are rural people and live there since birth. Sanmenxia (N 34.77; E 111.20; altitude: 376.08 m) in Henan province was built in the 1950s and also is a rural city. Residents in this new city partly immigrated from the whole of China, such as Northeast China and West China. In 2011, the pure annual income of rural people was 5601.40 CNY (about 889.11 USD) in Shanxi province and 6604.03 CNY (1048.26 USD) in Henan province, much lower than Beijing 14735.68 CNY (about 2339.00 USD) cited from China Statistical Yearbook 2012 [2]. Based on the Sixth National Census of China 2010 (http://www.stats.gov.cn/) [11] and the 2010 annual survey data of China Disabled Persons' Federation (http://www.cdpf.org.cn) [12], cataract surgical rate (CSR) was calculated as in Table 1.

Any patients who wanted to have the charge-free cataract operations on LEHET registered at the base hospital (a local hospital selected by the office of LEHET). After the systemic and ocular examinations and signing the informed consent at the base hospital, the patients were sent to LEHET. Preoperatively on LEHET, all patients underwent a complete ophthalmological examination, that is, measurement of presenting visual acuity (VA) by means of Snellen charts (performed by the nurses from the base hospital), intraocular pressure evaluation (IOP) by noncontact tonometer (Canon TX-10/TX-F, Tokyo, Japan) by the trained nurses from PUPH, slit lamp examination (Topcon SL-1E, Tokyo, Japan), and fundus examination (90 Dioptre, Volk Optical, Mentor, OH) with dilated pupil by the ophthalmologists from PUPH. Corneal curvature by Auto-Keratometer (Nikon Speedy-K, Tokyo, Japan), axial length (AL) and B-scan by ultrasonic system (ODM-2100, MEDA, Tianjing, China), and corneal endothelial counting (CEC) by Specular Microscope (Topcon SP-3000P, Tokyo, Japan) were performed by the trained technicians from PUPH on the patients suitable for operation. The flatter (*K*1) and steeper corneal curvature (*K*2) were read directly from the Auto-Keratometer, and *K* was calculated as the average of *K*1 and *K*2. Corneal radius (CR) was calculated from the formula CR  (millimeter, mm) = 1000 × 0.3375/*K*  (Diopter, D). The SRK/T formula for normal or long axial length (AL more than 25.00 mm) and Hoffer Q formula for short axial length (AL less than 22.00 mm) were used to calculate the power of intraocular lens (IOL) and the estimated postoperative refractive errors were less than ±0.25 D except patients with high myopia. LEHET was equipped with Specular Microscope, SP-3000P, in the first half of year 2012; the patients of Zhoukou and part of Yuncheng had no CEC measurement.

Exclusion criteria for this study are as follows: age less than 20 years, AL equal to or more than 27.00 mm, and history of intraocular surgery.

The study was in accordance with the tenets of the Declaration of Helsinki and has been approved by the institutional review board of PUPH. Written informed consent was obtained from all patients.

### 2.2. Statistical Analysis

The Student *t*-test was used to compare age and chi-square test was used to compare the female ratio between the groups. A *p* value less than 0.05 was considered to be statistically significant. Statistical analysis was performed using Statistical Product and Service Solutions software (SPSS version 20.0, Armonk, New York, USA).

## 3. Results

The demographic characteristics of the three missions are shown in Table 2. Totally, 3828 cataract patients (3828 eyes) were enrolled in this study, including 1419 males and 2409 females (male : female = 1 : 1.70) and 1984 right eyes and 1844 left eyes. There were no statistically significant differences between missions preoperatively in age, gender, and eye operated on.

As in Table 2, average age of these cataract patients was 69.50 ± 8.05, which was 69.10 ± 8.41 for males and 69.74 ± 7.82 for females (*p* = 0.019), respectively. In detail, the average age was 68.55 ± 8.12 for males and 69.57 ± 8.07 for females in Zhoukou (*p* = 0.056), 69.02 ± 8.33 for males and 70.16 ± 7.50 for females in Yuncheng (*p* = 0.010), and 69.52 ± 8.64 for males and 69.48 ± 7.92 for females in Sanmenxia (*p* = 0.933). Although the average age of females is older than males totally, that of males and females was of no difference for Zhoukou and Sanmenxia, except that of females which was older than that of males in Yuncheng.

As shown in Tables 3, 4, and 5, not only for males or females, but also for total patients, the preoperative VA (LogMAR) of these three groups is as follows: Zhoukou > Yuncheng > Sanmenxia. The patients in Sanmenxia had the best preoperative VA, even in each gender, significantly.

As shown in Tables 3, 4, and 5, there was a statistically significant difference in preoperative IOP between the patients of Yuncheng and Zhoukou, Yuncheng and Sanmenxia. The males, females, and total patients of Yuncheng had lower preoperative IOP compared with those in Zhoukou or Sanmenxia.

**Figure 1:**
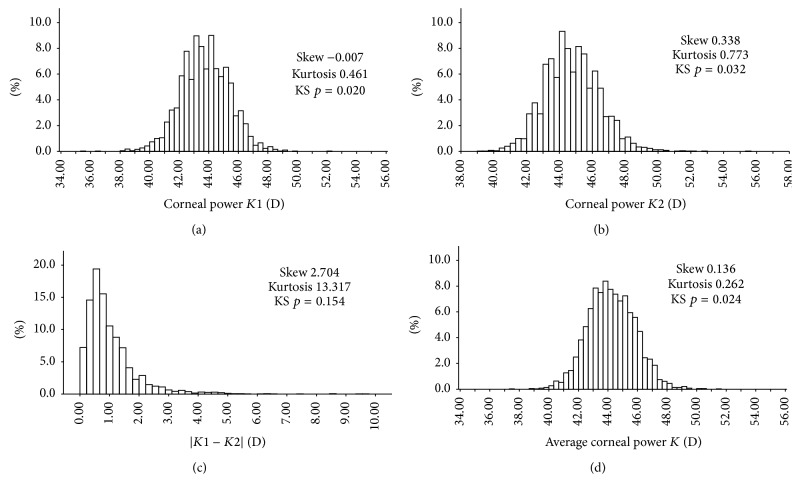
The distributions of corneal power in rural China. *K*1 (a), *K*2 (b), |*K*1 − *K*2| (c), and average corneal power (*K*) (d).

As shown in Figure 1 and Tables 3, 4, and 5, the patients of Zhoukou had lower *K*1 and *K*2, significantly. There was no statistically significant difference in *K*1 between those of Yuncheng and Sanmenxia, but *K*2 of Yuncheng was higher than Sanmenxia significantly. Respectively, both the males and females in Zhoukou had lower *K*1 and *K*2. However, for either the males or the females, there was no difference of *K*1 and *K*2 between those in Yuncheng and Sanmenxia.

Average corneal power (*K*) is an important parameter to calculate the power of IOL. In Figure 1 and Tables 3, 4, and 5, the patients in Zhoukou had lower average corneal power (*K*) significantly compared with the other two groups, the same for male and female patients in Zhoukou. But there was no significant difference in average corneal power (*K*) between those in Yuncheng and Sanmenxia, for either the males or the females.

The difference between *K*1 and *K*2 could be used to indicate the corneal astigmatism, which has the effect on the postoperative visual acuity. In Figure 1 and Tables 3, 4, and 5, the difference of *K*1 and *K*2 for the patients was as follows: Zhoukou > Yuncheng > Sanmenxia. That was the same for the females. But for the males except that |*K*1 − *K*2| of Zhoukou was higher than Sanmenxia significantly, there was no significant difference between Zhoukou and Yuncheng or between Yuncheng and Sanmenxia.

**Figure 2:**
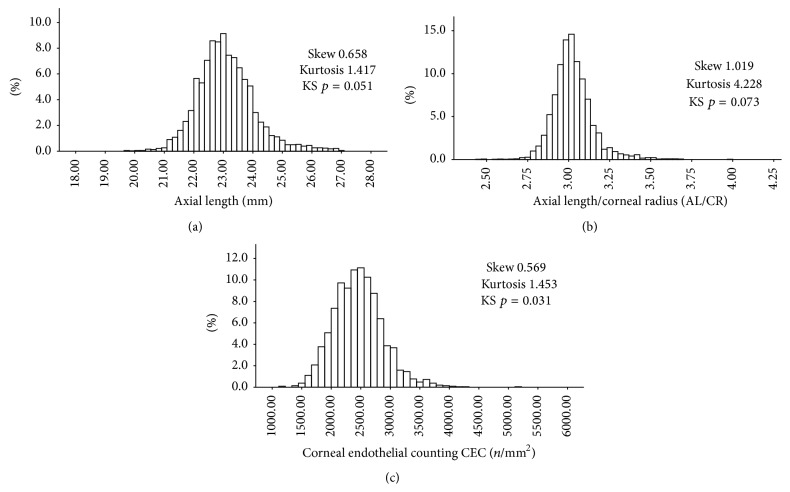
The distributions of axial length (AL), axial length/corneal curvature (AL/CR), and corneal endothelial counting (CEC) in rural China. Axial length (AL) (a), axial length/corneal curvature (AL/CR) (b), and corneal endothelial counting (CEC) (c).

AL is another important parameter to calculate the power of IOL. As seen in Figure 2 and Tables 3, 4, and 5, AL for the patients was as follows: Zhoukou < Sanmenxia < Yuncheng. For the males, AL of Zhoukou was shorter than the other two cities. For the females, AL of Yuncheng was longer than the other two sites. There was no significant difference in AL between Yuncheng and Sanmenxia for males or between Zhoukou and Sanmenxia for females.

The AL/CR ratio is highly correlated with the spherical equivalent as a previous study. As seen in Figure 2 and Tables 3, 4, and 5, the patients in Zhoukou had the smallest AL/CR ratio closer to 3.0, and Yuncheng and Sanmenxia had similar ratio. That is the same for the males and females.

CEC is a very important factor to decide the operation scheme and to predict prognosis. As there was no machine in Zhoukou at that time, we only could compare CEC between those in Yuncheng and Sanmenxia. As shown in Figure 2 and Tables 3, 4, and 5, CEC of Yuncheng was higher than Sanmenxia, which was same result for the males. But for the females, there was no significant difference in CEC between Yuncheng and Sanmenxia.

## 4. Discussion

This study explored the data of cataract patient, who had the free surgeries on LEHET, on ocular biometry of Chinese population in rural China. And our study provided the normative data on *K*1, *K*2, |*K*1 − *K*2|, average corneal power (*K*), AL, AL/CR, and CEC of this population; those were 43.74 ± 1.64 D, 44.75 ± 1.68 D, 1.02 ± 0.86 D, 44.24 ± 1.60 D, 23.04 ± 1.49 mm, 3.03 ± 0.12, and 2462.36 ± 423.65/mm^2^, respectively.

Our study showed AL in rural Chinese population was normally distributed with a positive skew and a big kurtosis (1.417). Skew and kurtosis have been reported in the distribution of AL in the Reykjavik Eye study [13], the Singapore Malay Eye study [7], the Singapore Indian Eye study [10], and Fotedar et al.'s study [14]. Hence, this is the first report of the appearance of big kurtosis in the distribution of AL in rural Chinese population.

It is worthwhile comparing our findings with those of the Tanjong Pagar study on adult Chinese population in Singapore, which also used A-scan. The mean AL in that study (23.23 ± 1.17 mm) was a little longer than in our study (23.04 ± 1.49 mm). Moreover, AL in our study is shorter than Latinos (23.38 mm) in Los Angeles with A-scan [8], Malay people (23.55 mm) [7] in Singapore, Indian people (23.45 mm) [10] in Singapore, and Caucasian people (23.44) [14] in the Blue Mountains area in Australia with IOLMaster, longer than another Asian population (22.76 mm) in Myanmar with Ocuscan [9]. The similarity of AL in those studies with A-scan and ours is likely to be explained by the same method of AL measurement. The difference in AL of these studies might be explained by a greater degree of urbanization in Singapore and subsequently a higher rate of axial myopia [10]. Those three studies with IOLMaster indicated that the race might have significant effect on AL compared with region as the similarity of Indian and Caucasian people.

The corneal power *K*1, corneal power *K*2, and *K* (average corneal power) in our study were not normally distributed with different skews and kurtosis. That is similar to the Singapore Malay Eye study [7] and Fotedar et al.'s study [14]. On the contrary, |*K*1 − *K*2| in our study was normally distributed with a positive skew (2.704) and a significant kurtosis (13.317). Moreover, the preoperative visual acuities in the three missions of our study had the same trend as |*K*1 − *K*2|, both of that of males and females are the same. It indicated that the corneal astigmatism might have obvious effect on the visual acuity.

There is evidence that the AL/CR ratio of an emmetropic eye is usually very close to 3.0, and a higher AL/CR ratio was reported to be a risk factor in myopia [15, 16]. However, few studies have reported the AL/CR ratio [10]. Compared with Zhoukou, the patients in Yuncheng and Sanmenxia had similar AL/CR ratio, also in males and in females. The Singapore Indian Eye study [10] showed that the AL/CR ratio correlated more highly with the spherical equivalent than AL alone. This correlation indicated that longer eyes are not necessarily myopic and worse presenting visual acuity, including those that are long because of overall body stature. The patients in Zhoukou, who had shorter AL and AL/CR closer to 3.0, had the worst preoperative visual acuities. This indicated that in rural Chinese population at least in the cataract patients the AL/CR ratio, in other words, the spherical equivalent, had less effect on the visual acuity than |*K*1 − *K*2|, the corneal astigmatism.

In conclusion, this study provides normative ocular biometry in a large, representative rural Chinese population. The AL is normally distributed with a positive skew and a big kurtosis. The corneal power *K*1, corneal power *K*2, and *K* (average corneal power) are not with normal distribution. The corneal astigmatism might have a significant effect on the visual acuity.

## Figures and Tables

**Table 1:** CSR of China 2010.

Province	Population	Cataract surgeries	Charge-free cataract surgeries for low-income people	CSR	Charge-free CSR	Ratio of charge-free cataract surgeries for low-income people
Beijing	19,612,368	11961	1402	610	71	11.72%
Shanxi	35,712,101	15902	4929	445	138	31.00%
Henan	94,029,939	36935	9915	393	105	26.84%

CSR: cataract surgical rate.

**Table 2:** Demographic characteristics of the three groups.

	Zhoukou (*n* = 991)	Yuncheng (*n* = 1240)	Sanmenxia (*n* = 1497)	*p*
Age (years)	69.20 ± 8.10	69.73 ± 7.84	69.49 ± 8.20	0.293

Sex Male/female	355/636	502/838	562/935	0.640

Eye operated on Right/left	538/453	697/643	749/748	0.113

*p* values were calculated with ANOVA.

**Table 3:** Biological parameters of the three groups.

		Zhoukou	Yuncheng	Sanmenxia	Total	*p*
Preoperative visual acuity	LogMAR	1.20 ± 0.38	0.95 ± 0.37	0.63 ± 0.43	0.87 ± 0.46	0.000
	Less than 6/60 *n* (%)	763 (76.99%)	653 (48.73%)	636 (42.48%)	2052 (53.61%)	0.000
	Equal to or better than 6/60 and less than 6/18 *n* (%)	218 (22.00%)	565 (42.16%)	710 (47.43%)	1493 (39.00%)	0.000
	Equal to or better than 6/18 *n* (%)	10 (1.01%)	122 (9.10%)	151 (10.09%)	283 (7.39%)	0.000

Preoperative IOP (mmHg)		14.53 ± 3.44	13.98 ± 2.92	14.78 ± 3.11	14.44 ± 3.15	0.000

Corneal curvature (D)	*K*1	43.40 ± 1.65	43.89 ± 1.68	43.82 ± 1.56	43.74 ± 1.64	0.000
	*K*2	44.59 ± 1.79	44.88 ± 1.70	44.75 ± 1.57	44.75 ± 1.68	0.000
	|*K*1 − *K*2|	1.20 ± 1.03	0.98 ± 0.81	0.93 ± 0.76	1.02 ± 0.86	0.000
	Average corneal power (*K*)	44.00 ± 1.64	44.38 ± 1.64	44.29 ± 1.52	44.24 ± 1.60	0.000

Axial length (AL) (mm)		22.95 ± 1.05	23.17 ± 0.95	23.12 ± 0.92	23.04 ± 1.49	0.000

AL/CR		2.99 ± 0.14	3.04 ± 0.12	3.03 ± 0.11	3.03 ± 0.12	0.000

CEC (*n*/mm^2^)		*∗*	2505.63 ± 431.98^*∗*^	2445.24 ± 419.23	2462.36 ± 423.65^*∗*^	0.003

*p* values were calculated with ANOVA or chi-square test.

^*∗*^LEHET was equipped with Specular Microscope, SP-3000P, in the first half of year 2012; the patients of Zhoukou and part of Yuncheng had no CEC measurement.

**Table 4:** Biological parameters for males of the three groups.

	Zhoukou	Yuncheng	Sanmenxia	Total	*p*
Preoperative visual acuity (LogMAR)	1.21 ± 0.38	0.98 ± 0.36	0.66 ± 0.44	0.89 ± 0.46	0.000
Preoperative IOL (mmHg)	14.28 ± 3.46	13.69 ± 2.87	14.50 ± 3.18	14.16 ± 3.17	0.000
Corneal curvature (D)					
*K*1	42.80 ± 1.52	43.37 ± 1.64	43.31 ± 1.44	43.20 ± 1.55	0.000
*K*2	43.86 ± 1.50	44.27 ± 1.64	44.19 ± 1.45	44.13 ± 1.54	0.000
|*K*1 − *K*2|	1.07 ± 0.85	0.91 ± 0.80	0.87 ± 0.66	0.94 ± 0.77	0.001
Average corneal power (*K*)	43.33 ± 1.45	43.82 ± 1.59	43.75 ± 1.41	43.67 ± 1.49	0.000
Axial length (AL) (mm)	23.12 ± 0.90	23.44 ± 0.89	23.46 ± 0.88	23.37 ± 0.90	0.000
AL/CR	2.97 ± 0.12	3.04 ± 0.11	3.04 ± 0.11	3.02 ± 0.11	0.000
CEC (*n*/mm^2^)	*∗*	2537.49 ± 450.38^*∗*^	2437.86 ± 439.39	2467.32 ± 444.71^*∗*^	0.004

*p* values were calculated with ANOVA.

^*∗*^LEHET was equipped with Specular Microscope, SP-3000P, in the first half of year 2012; the patients of Zhoukou and part of  Yuncheng had no CEC measurement.

**Table 5:** Biological parameters for females of the three groups.

	Zhoukou	Yuncheng	Sanmenxia	Total	*p*
Preoperative visual acuity (LogMAR)	1.19 ± 0.38	0.93 ± 0.37	0.61 ± 0.42	0.85 ± 0.46	0.000
Preoperative IOL (mmHg)	14.67 ± 3.43	14.17 ± 2.93	14.95 ± 3.06	14.60 ± 3.13	0.000
Corneal curvature (D)					
*K*1	43.74 ± 1.63	44.20 ± 1.63	44.13 ± 1.54	44.05 ± 1.61	0.000
*K*2	45.00 ± 1.81	45.24 ± 1.63	45.09 ± 1.54	45.12 ± 1.65	0.016
|*K*1 − *K*2|	1.27 ± 1.11	1.02 ± 0.82	0.96 ± 0.81	1.06 ± 0.91	0.000
Average corneal power (*K*)	44.37 ± 1.63	44.72 ± 1.58	44.61 ± 1.49	44.58 ± 1.56	0.000
Axial length (AL) (mm)	22.86 ± 1.11	23.01 ± 0.96	22.91 ± 0.88	22.93 ± 0.97	0.008
AL/CR	3.00 ± 0.15	3.05 ± 0.12	3.03 ± 0.11	3.03 ± 1.29	0.000
CEC (*n*/mm^2^)	*∗*	2484.59 ± 418.72^*∗*^	2449.66 ± 406.84	2459.30 ± 410.29^*∗*^	0.174

*p* values were calculated with ANOVA.

^*∗*^LEHET was equipped with Specular Microscope, SP-3000P, in the first half of year 2012; the patients of Zhoukou and part of  Yuncheng had no CEC measurement.
